# Seed Bank Conservation and Incipient Seed Development in Orchids Colonizing Mining Wastes: Results of a Field Pilot Experiment

**DOI:** 10.3390/plants11233315

**Published:** 2022-12-01

**Authors:** Antonio De Agostini, Donatella Cogoni, Annalena Cogoni, Andrea Vacca, Giuseppe Fenu, Pierluigi Cortis

**Affiliations:** 1 Department of Life and Environmental Sciences, University of Cagliari, 09123 Cagliari, Italy; 2 Department of Chemical and Geological Sciences, University of Cagliari, 09042 Monserrato, Italy

**Keywords:** *Epipactis tremolsii*, mining areas, heavy metals, germination, translocation, seed packet, soil pollution

## Abstract

As they represent actual or potential risks to human and environmental safety and health, abandoned mines are a major global problem. The heavy metal-polluted tailings dump of Barraxiutta (Domusnovas, southwestern Sardinia, Italy) is home to a metallicolous population of *Epipactis tremolsii* (Orchidaceae). A reclamation of the abandoned mine area seems to be approaching, and such an intervention may pose a serious risk for the maintenance of the unique orchid population colonizing the mine wastes. In the present work, the seed packet technique was implemented for the first time to observe orchid seed development in mine wastes. This approach allowed us to explore different seed-based conservation options for the metallicolous orchid population and to gain a deeper grasp of population dynamics and ecology. Four different sowing treatments were set up in the tailing dump and in a near unpolluted site (control site). The field phase of the experiment lasted for 10 months, a period in which the experimental seed bank preservation and incipient seed development were observed and statistically approached. Our findings observed no significant seed loss happening during the experiment, demonstrating the suitability of the seed packet technique to also explore seed bank conservation and development in extreme environmental conditions (i.e., polluted mine wastes). This field method will be a useful tool to further explore the more effective translocation and quasi in situ conservation alternatives for the *E. tremolsii* metallicolous population. Incipient and site-specific seed development (non-mycorrhizal stage) was observed during the experiment. A plant–soil fungus interaction at the seed level was also observed, the nature of which remains to be ascertained in further studies providing a longer duration for the field phases.

## 1. Introduction

Mining has been considered one of the principal sources of environmental pollution worldwide [1,2,3]. Besides the impact mines have on local and regional environments during the production phase, they may continue to degrade surrounding land, water bodies and air even after their closure and abandonment [4]. The management of abandoned mines is challenging yet urgent, representing an unresolved environmental problem [1,2,3,4,5]. The reclamation of abandoned mines could, however, endanger the conservation of peculiar and rare metallicolous flora settling there. In fact, despite heavy metals being known for their detrimental effects on the biosphere, some plants (metal-tolerant or metallophyte *taxa*) have evolved to thrive despite their occurrence [6]. The peculiar biota present in natural or anthropogenic metalliferous areas should be preserved and studied to investigate those mechanisms of adaptation that allow them to live in such harsh contexts [7,8].

Orchids can often be found growing in naturally metalliferous environments [9,10] as well as in disturbed, anthropogenically polluted habitats, including mine wastes [11,12,13]. The Barraxiutta tailing dump (Sardinia, Italy) makes no exception in this sense since it hosts a population of *Epipactis tremolsii* Pau (Orchidaceae) counting approximately 500 individuals. The Barraxiutta tailing dump is contaminated by Pb, Zn and Fe (among the others) and is formed by fine particles [14,15]. Given the features of the area, its reclamation appears urgent and more and more imminent (2020–2024 according to the Sardinian Industrial Plan guidelines). Previous studies on the metallicolous orchid population described the orchids settling there bioaccumulating and translocating soil pollutants in their organs. As a result, plants have reduced photosynthetic efficiency and, despite being smaller, produce bigger and heavier seeds [14,15]. Despite the extreme ecological growing context, orchids complete their biological cycle, reaching the flowering stage, and producing fruits and thousands of viable seeds per plant each year [14].

Given this framework, the following ecological issue delineates: a reclamation of the Barraxiutta tailing dump would inevitably modify the ecological features of the site, posing a serious threat to the maintenance of the *E. tremolsii* metallicolous population, its genetic heritage and its potential as an evolutionary model. To prevent and mitigate any negative outcome resulting from the reclamation of the tailing dump, it is necessary to find practical methods that will allow for the characterization of the population-specific dynamics behind the maintenance of the metallicolous orchid population. The objectives of the present work were therefore as follows: (i) to test the suitability of the seed packet technique for exploring orchid seed bank conservation and development in the extreme environment of the mining waste dump and (ii) to explore different conservation options (based on seed translocation) to integrate the reclamation of the tailing dump in order to preserve the metallicolous orchid population settling there.

The seed packet technique allows us to observe germination dynamics in situ, even in small or microscopic seeds (such as orchid seeds) [16]. This technique, conceived and described in [16], consists of burying a known number of seeds enclosed in tight-meshed net packets to be then retrieved and observed. Packets allow for an easy retrieval of the seed material and protect seeds from soil microfauna while allowing seed–soil microbiome interactions that are mandatory for any germination event in orchids to take place.

The validation of this technique in such an extreme environment would provide a valuable tool in the conservation of the Barraxiutta metallicolous orchid population and in similar conservation issues. An appropriate knowledge regarding the species-specific and population-specific recruitment mechanisms and dynamics is in fact decisive in the conservation of endangered plants populations, and several conservation approaches have failed because of a lack of knowledge [17,18,19]. To address the aims of this study, experimental *E. tremolsii* seed bank preservation and seed early developmental stages were followed during approximately a year (10 months) in a seed packet experiment (full crossed design) which was carried out in the polluted tailing dump and in an unpolluted control site nearby. We hypothesized that the technique would prove to be valid, even in the extreme study area, and that the two studied populations would show different seed development dynamics given the extreme soil pollution featuring one of them.

## 2. Results

### 2.1. Seed Conservation during the Experiment and Morphometric Parameters

**Figure 1:**
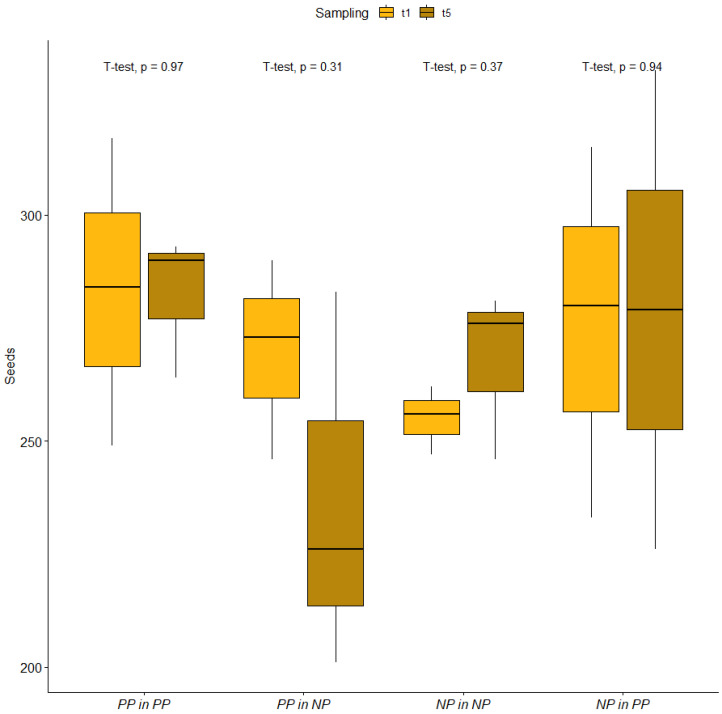
Comparison between the number of seed collected in the first (t1) and in the last (t5) retrieval campaigns. First and last retrieval campaigns (t1 and t5, respectively) are indicated by different colours. Number of seeds is reported on the *y* axis and sowing treatments are reported on the *x* axis (PP in PP indicates metallicolous seeds sowed in the tailing dump; PP in NP indicates metallicolous seeds sowed in the control site; NP in NP indicates control seeds sowed in the control site; NP in PP indicates control seeds sowed in the tailing dump). The results of *t* tests are reported above each series of boxplots as *p* values. Level alfa at 0.05. Each boxplot reports 50% of the measured values (inside the box), comprised between the first quartile value (lower side of the box) and the third quartile value (upper side of the box). The median is indicated by the black line inside the box, while whiskers join the first and third quartiles with lower and higher measured values, respectively (if present, outliers are reported as black dots).

Seed content in each packet after the retrieval ranged from 201 to 359 seeds, SE = 4.56 (detailed data on the number of retrieved seeds are reported in Table S1), and no difference was observed between t1 and t5 in no sowing treatment (Figure 1, Table S2).

**Figure 2:**
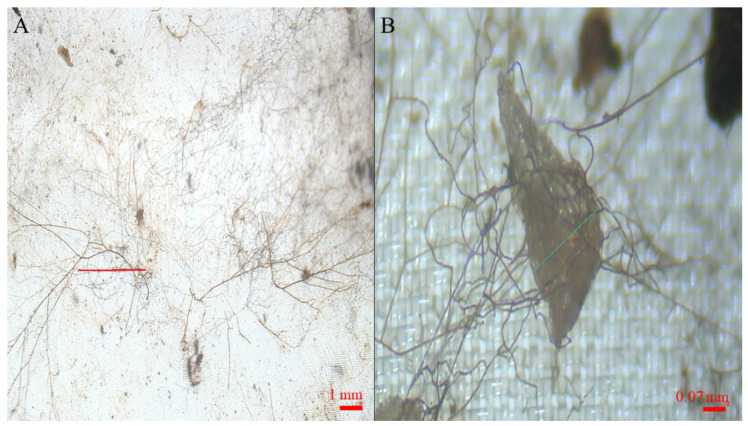
Stereomicroscope images of fungal hyphae invading the packets. Panel (**A**): hyphae in the packet. Panel (**B**): hyphae and a developing (S2) control seed retrieved from the control site.

**Figure 3:**
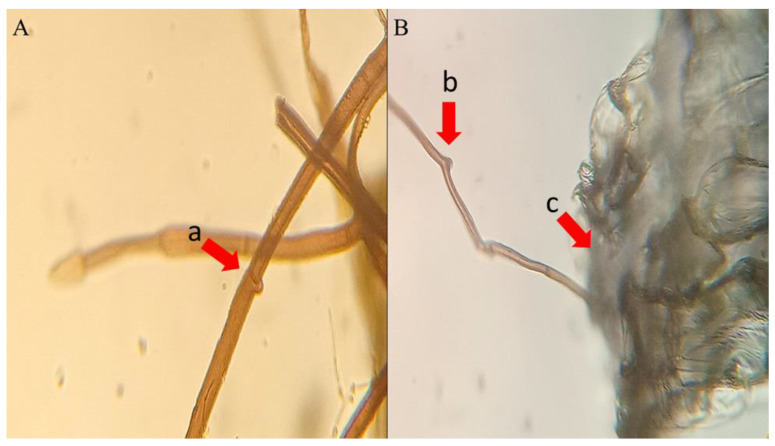
Fungal hyphae (400× magnified in panel (**A**), 100× magnified in panel (**B**)). In panel (**A**), the hypha clamp connection is shown (a). In panel (**B**) a fungal hypha (b) enters a *E. tremolsii* seed through its operculum (c).

The ANOVA and GLM approaches confirmed a non-significant effect of time on the seed count in any of the sowing treatments. The higher number of seeds at t2 and t3 in the NP in NP sowing treatment should be attributed to flaws in the packets building. ANOVA and GLM results are extensively reported as supplementary material (Figure S1 and Tables S3 and S4). Starting from t1 packet observation at the stereo microscope permitted the observation of dense networks of fungal hyphae invading the packets (Figure 2A and Figure 3A), as well as of incipient seed–soil fungi interaction (Figure 2B and Figure 3B) in packets retrieved from the contaminated and control site.

**Figure 4:**
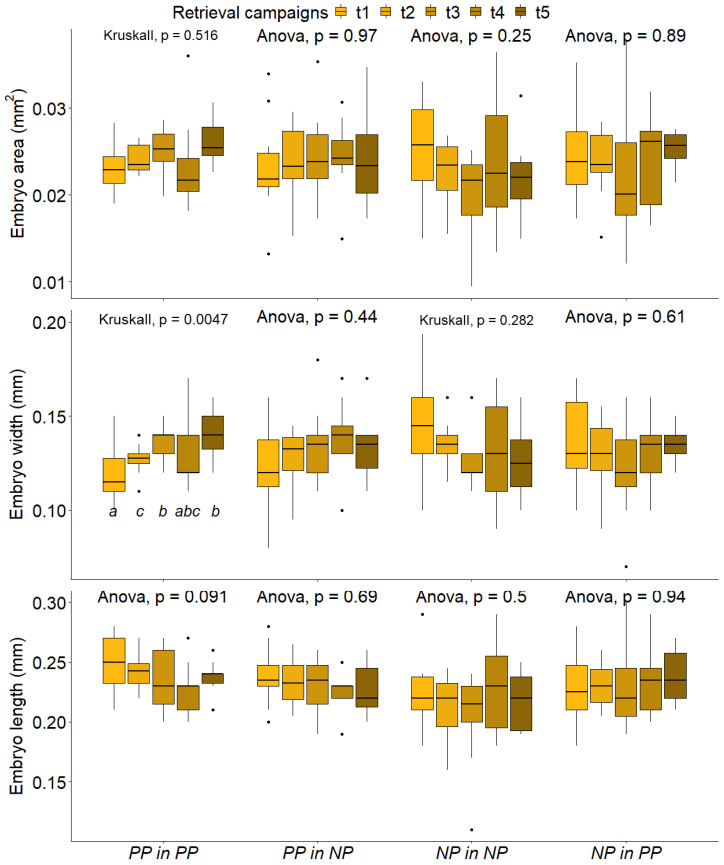
Analysis of variance results on morphometric parameters measured in the different retrieval campaigns and in the different sowing treatments. Retrieval campaigns from t1 to t5 are indicated by different colours. Sowing treatments are reported on the *x* axis and morphometric parameters values are reported on the *y* axis, (PP in PP indicates metallicolous seeds sowed in the tailing dump; PP in NP indicates metallicolous seeds sowed in the control site; NP in NP indicates control seeds sowed in the control site; NP in PP indicates control seeds sowed in the tailing dump). ANOVA or Kruskall–Wallis test results are reported above each series of boxplots as *p* values. Post hoc results are reported by compact letter display under each boxplot (the same letters shared by different plots indicating the absence of significant statistical differences). Level alfa at 0.05. Each boxplot reports 50% of the measured values (inside the box), comprised between the first quartile value (lower side of the box) and the third quartile value (upper side of the box), the median is indicated by the black line inside the box, while whiskers join the first and third quartiles with lower and higher measured value, respectively (if present, outliers are reported as black dots).

An analysis of variance in morphometric parameters showed that the only sowing treatment in which parameters varied among the retrieval campaigns was the non-mixed sowing treatment PP in PP (Figure 4 for embryo size and Figure S2 for coat size). More precisely: embryo width significantly increased by 8.3% (t2 with respect to t1), 14.3% (t5 with respect to t2) and 21.4% (t5 with respect to t1); coat area significantly increased 11.54% (t3 and t5 with respect to t4); coat width significantly increased 24.25% (t3 with respect to t1) and 21.22% (t3 with respect to t4). In summary, embryo width and coat width and area increased with time, reaching a peak at t3. Morphometric data and analysis of variance results are extensively reported in Tables S5 and S6.

### 2.2. Developing Embryos

During the experiment it was possible to observe, isolate and measure eleven S2 seeds. S2 seeds were observed at t3 and t4, 10 were viewed in non-mixed sowing treatment (NP in NP and PP in PP), and one in a mixed sowing treatment (NP in PP). S2 seeds were observed in 40% of packets in the NP in NP sowing treatment and in 6.67% of packets in PP in PP and NP in PP. No packets in the PP in NP sowing treatment contained S2 seeds. S2 seeds were significantly larger if compared to the rest of the undeveloped seeds (one-sample test results are reported in Table S7). More precisely, in the S2 seeds with respect to S1 unmodified seeds: embryo area increased 37.5% and embryo width increased 38.64% in PP in PP at t3; embryo area increased 71.43% in NP in NP at t3; embryo area increased 40%, embryo width increased 40%, embryo length increased 8% and coat width increased 24.33% in NP in NP at t4; embryo area increased 20%, embryo width increased 26.7% and coat width increased 30.77% in NP in PP at t4.

## 3. Discussion

Species belonging to the *Epipactis* genus are reported to colonize polluted environments and abandoned mines [11,12,13,20,21]. Nevertheless, to the best of our knowledge, the population here studied is the only in Sardinia settling on polluted mining wastes. Due to the public health issues this can cause, the abandoned mining dump of Barraxiutta may be subjected in a near future to remediation- and restoration-oriented interventions. Given this scenario, conservationists approaches and quasi in situ conservation options may be required in the near future for preservation of the unique *E. tremolsii* metallicolous population. This study reports the results of a pilot experiment carried out to evaluate the feasibility of the seed packet technique to explore population dynamics and translocation options for the metallicolous orchid population.

Translocation could in fact represent a way to maintain endangered populations [18,22,23], such as the orchid population here studied. Translocation, or the quasi in situ conservation *sensu* [24], are based on the controlled placement of plant material into a (semi-) natural area to preserve the genetic diversity of peculiar, endangered populations [22,25]. Similar approaches to plant conservation are increasingly prevalent worldwide [26,27,28]. Nevertheless, the translocation of orchids should take into account the importance of specific germination niches for the intervention to be successful [22,25]. Germination of orchid seeds requires a complex of abiotic and biotic factors called a germination niche [29,30]. As a consequence, the conservation and management of endangered orchid populations via translocation results in a challenging task in which the knowledge of the species-specific and population-specific germination and recruitment mechanisms is crucial [17,18,29]. Given this scenario, the seed packet technique proved to be suitable to explore incipient seed development in the study area since the seed material was preserved during the experiment, and any variation in seed morphometry was promptly observed and related to a sowing treatment.

Long-lived seed banks and delayed germination are typical features of forest orchids such as *Epipactis* [31,32,33] and were observed in the present study too. The seed packet-based experiment carried out in the present study in a polluted tailing dump provided a unique look at the resistance of *E. tremolsii* seeds to extreme abiotic stressors. In fact, *E. tremolsii* seeds, both from metallicolous and non-metallicolous populations surrounding the tailing dump, remained unaltered despite heavy metals, low pH levels, and no vegetal coverage nor litter protecting the seed bank. The good permanence of non-metallicolous *E. tremolsii* seeds in the mining waste is an important element for conservation purposes since it suggests how surrounding non-metallicolous populations could implement the seed bank in the mining dump. Moreover, future field experiments exploring seed translocation options should not consider the issue of seed loss in the tailing dump extreme environment.

Beside seed bank conservation, our study aimed to explore early seed development in a full crossed experimental design to test for site-specific seed development dynamics. To anticipate the remediation of the study area, the field phase of the experiment was set up to 10 months. Due to the duration of the field phase, no mycorrhizal stage was observed in the sowed seeds. Thus, we proceeded to evaluated seed development by morphometry. Seed morphometry may be affected by the availability of nutrients, light and peculiar growth conditions affecting mother plants [15,29] as well as by the position and number of capsules in the inflorescence and by differences in the timing of flower pollination [34,35]. In the specific study case, seeds produced in the tailing dump are characterized by larger coats and fixed-sized embryos when compared with seeds produced by non-metallicolous populations [15]. Nevertheless, the significant variations in seed morphometry that were observed in the present study should be attributed to the sowing treatment, since seed material was sampled to avoid the factors listed above to confound experimental results.

Soil moisture positively affects the development of the seeds in orchids [29]. Coherently, in the present study embryos began to swell when soil moisture in the study area reached a peak. More precisely, starting from the third retrieval campaign (t3), embryos increased in width and coats increased in width and area. Considering potential evapotranspiration and rainfall data for the study area, it was found that t3 was the retrieval campaign featured by the moistest soil. Moister soils could have determined seed imbibition, i.e. the first mandatory step to triggering germination. Coherently, starting from t3 seeds started to switch from S1 to the S2 developmental stage, meaning a shift from unmodified seed to incipient and non-mycorrhizal seed development [32].

Coherently with our initial hypothesis, in the present study seed development occurred almost exclusively in non-mixed sowing treatments, that means control seeds sown in the control site and seeds from the tailing dump sown in the tailing dump. Incipient seed development occurring mainly in non-mixed sowing treatment suggests the existence of site-specific seed development dynamics, determined by the peculiar abiotic features of the substrate in the two studied populations. In fact, despite orchid germination niches are mostly determined by the occurrence and abundance of symbiotic soil fungi, incipient seed development in orchids is mostly triggered by abiotic factors [32]. Given what has been said so far, metallicolous *E. tremolsii* may struggle to settle outside of the tailing dump, and vice versa, non-metallicolous seeds could struggle to settle on the tailing dump due to the extreme abiotic stressors present there. For this reason, the reclamation of the Barraxiutta tailing dump could have catastrophic outcomes in terms of the conservation of the metallicolous orchid population.

The present study proved the viability of the seed packet technique to gain valuable insights into germination dynamics in the study area and highlighted differential and site-specific seed development. Future studies aiming to increase knowledge of germination dynamics and to explore translocation and quasi in situ conservation options in the metallicolous orchid population should provide for a longer duration of germination experiments and for a wider coverage of the study area.

In conclusion, in the complex ecological and public health scenario here presented, solid knowledge of germination dynamics of the *E. tremolsii* metallicolous population needs to be further acquired to inform any future intervention in the mining dump of Barraxiutta.

## 4. Materials and Methods

### 4.1. Plant Species

*Epipactis tremolsii* Pau (syn. *E. helleborine* subsp. *tremolsii* (Pau) E.Klein. for other authors) is a perennial rhizomatous orchid that frequently occurs in dense woodlands edges and forest clearings up to 900 m a.s.l. The species thrives in mid-shady to shady contexts on mature soils. During the vegetative season (middle spring), *E. tremolsii* plants produce one to six aerial stems up to 60 cm tall [36] and inflorescences hosting up to 50 cross-pollinating flowers. In Sardinia, the flowering season occurs in late spring [36]. The studied species is not subjected to any protection or evaluation by the IUCN (International Union for Conservation of Nature and Natural Resources) besides the CITES (Convention on International Trade in Endangered Species of Wild Fauna and Flora).

### 4.2. Contaminated and Control Site

**Figure 5:**
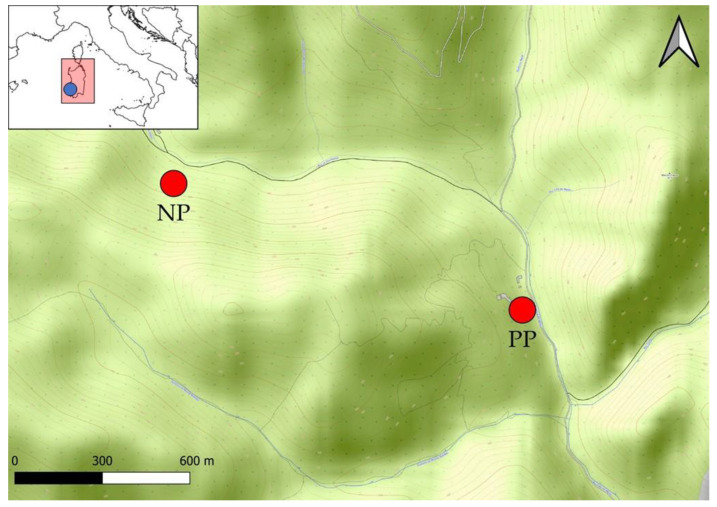
Map of the study area. NP and PP indicates the control and the metallicolous *E. tremolsii* population, respectively. The blue dot indicates the study area in Sardinia.

This study was carried out in the field. Seeds were sampled and sowed in the tailing dump *E. tremolsii* population (polluted population, PP, hereafter) and in a control *E. tremolsii* population not affected by mining pollution (natural population, NP, hereafter). The Barraxiutta mining dump (Domusnovas, Sardinia, Italy) covers c. 3000 m^2^ and originates from the flotation process of Galena and Sphalerite. As a result, high contents of Pb (up to 5 mg g^−1^), Zn (up to 13 mg g^−1^) and Fe (up to 56 mg g^−1^) were measured in the dump (more extensive data on soil pollution are reported as supplementary data in Table S8). The mining by-products in the dump are formed by fine particles and the plant coverage is almost absent there [14,15]. NP is located near (around 1 Km as crow flies) the Barraxiutta tailing dump, and the growth conditions between the two populations are comparable except for soil features (Figure 5). In fact, both populations settle in forest clearings. However, in the tailing dump, the soil is sandy, poor in organic matter and extremely polluted by heavy metals. Conversely, in the control site, the soil is mature and rich in organic matter while heavy metal pollution is absent. NP hosts c. 50 adult individuals, while in PP c. 500 adult individuals were counted. For a more extensively detailed PP and NP description, refer to [14,15].

Evapotranspiration (the ratio between precipitation and temperatures) and rainfall (total of precipitations) data in the study area (Table S9) were obtained from the database of the climatic monitoring authority of Sardinia [37], and both were used to interpret seed development observed during the experiment on the basis of environmental humidity.

### 4.3. Seed Collection and Seed Packet Construction

Seeds were collected from PP and NP on the same day (23 July 2020). Mature fruits were collected and individually preserved in 1.5 mL Eppendorf tubes. To ensure the seed samples were representative of the genetic diversity of the two populations, 10 plants per population were randomly selected and two fruits per plant were collected (20 fruits sampled in each population), following the recognized national protocol [38], adapted to small populations of rare species [39]. In the laboratory, seeds from the two populations were cleaned, excerpted from the fruits, and carefully evaluated by microscopy. Seed vitality was assessed by checking for intact and well-developed embryos, and by this PP and NP seed vitality. The results were comparable and over the 95% in both populations. Seed packets were prepared as described in [16] (see Figure S3 for details), with only minor modifications. Squares of 5 cm × 5 cm 20 µm mesh size plankton net (SCUBLA S.r.l., Remanzacco, Italy) were framed in 24 mm × 36 mm glassless plastic slides mounts (GEPE Geimuplast GmbH, Farchant, Germany). A total of 60 packets were prepared and an identification code was reported on each one. By using a laboratory micro spoon of 0.05 mm^3^, a consistent and known (estimated) number of seeds was enclosed in each packet. The mean number (±standard error, SE) of PP and NP seeds in each packet was estimated through 5 repeated counts. As a result, PP packets contained c. 240.40 ± 6.81 (mean ± SE) seeds, while NP packets contained c. 286.00 ± 6.88 (mean ± SE) seeds.

### 4.4. Experimental Design

**Figure 6:**
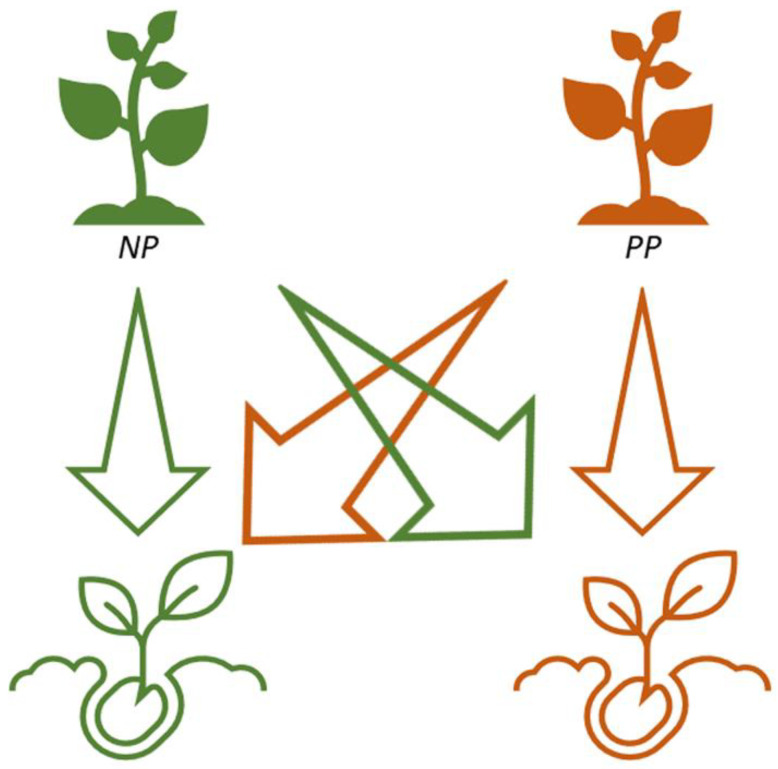
Experimental design scheme. NP and PP indicates the control and the metallicolous *E. tremolsii* population, respectively.

PP and NP seed packets were sown in a full crossed experimental, design resulting in four sowing treatments: NP in NP, NP in PP, PP in PP, PP in NP (Figure 6). In each sowing site, 15 NP and 15 PP packets were buried in experimental parcels of 5 m × 5 m, in horizontal position at a depth of about 5 cm. Packets were secured to a camping peg in groups of five by mean of a fishline. Seed development was monitored during a 10-month experiment. Packets were buried in the month of July (on 2 July 2020), when *E. tremolsii* seed dispersion naturally occurs in the wild and four months passed before the first packet retrieval, in accordance with the slow germination dynamic that features forest orchids such as *E. tremolsii*. Packet retrieval started in November 2020 and was divided into five retrieval campaigns (t1 to t5), each separated by 45 days. During each retrieval campaign, 12 packets were collected (three packets per sowing treatment as replicates). Packet retrieval ended in May 2021 (on 12 May 2020).

### 4.5. Seeds Observation and Measurements

After each retrieval campaign, the packets were transported to the laboratory under controlled conditions (in the dark and at constant humidity) and all observations were conducted within two days. Seed analysis was carried out by mean of a stereomicroscope connected to a high-definition camera (TiEsseLab TrueChrome HD IIS, Tiesselab, Milano, Italy) and implemented by a measurement software (TiEsseLab IS CAPTURE Rel. 3.6.7, Tiesselab, Milano, Italy). To detect any significant seed loss, the number of intact seeds in each sowing treatment was compared with that of the previous retrieval campaign. Seeds were then assigned to a developmental stage according to the scheme reported in [32], from S1 (unmodified seed) to S2 (swollen, non-mycorrhizal seedling) (Figure S4). Whenever a seed showed early signs of development, represented by a marked swelling, it was measured and photographed (in Figure S5 some of the seeds we assigned to S2 developmental stage compared with a S1 unmodified seed). As reported in [15], seeds were measured via the following parameters: coats and embryos width, height, and area. Any swelling seeds plus 10 unmodified seeds per packet were measured in each retrieval campaign.

### 4.6. Statistical Analysis

Seed loss during the field phase of the experiment was assessed by comparing seed number at t1 and t5 by the use of a *t* test in each sowing treatment. A focus on seed number variations in each retrieval campaign was obtained by the analysis of variance (ANOVA), a process which was conducted on the number of seeds retrieved in each retrieval campaign in the different treatments. ANOVA was performed after checking for its assumptions. A post hoc Tukey’s test was performed when necessary. ANOVA results were later compared with the results of the GLM quasi-Poisson regression applied to seed count data, with time being considered as an explanatory variable of seed count (dependent variable). Changes in seed morphological traits during the experiment were tested by ANOVA or Kruskall–Wallis analysis of variance depending on whether or not ANOVA assumptions were satisfied. Post hoc Tukey’s or Wilcoxon signed-rank tests were performed depending on whether ANOVA or Kruskall–Wallis analysis of variance were used. Post hoc results were represented in the plots by using the compact letter display (the same letters shared by different plots indicating the absence of significant statistical differences). One-sample *t* tests or one-sample Mann–Whitney U tests (depending on applicability criteria) were both used to test S2 seeds against the S1 seeds collected in the same retrieval campaign and sowing treatment (*p* values were adjusted for multiple testing by Bonferroni correction). All statistical analyses were carried out using the 1.2.1335 version of the R-Studio software [40] (implemented by “ggpubr” package [41]).

## 5. Conclusions

The results reported in this study were obtained by implementing the seed packet technique for the first time to explore the seed bank conservation and incipient seed development of an orchid (*E. tremolsii*) in the extreme environment of an abandoned mining dump. Population-specific seed development dynamics were observed though the brief duration of the field phase of the study permitted to describe only the first, non-mycorrhizal stage of the seed development process. If the site specificity would be further proved also in the mycorrhizal stages of seed development, this will need to be taken into account to set up the better conservation and quasi in situ conservation options for the metallicolous orchid population. The implementation of the seed packet technique in extreme environments, such as mine wastes, could provide unique and crucial insights into the role of fungal symbiosis in the colonization of harsh and extreme environments by orchids.

## Data Availability

Not applicable.

## Supplementary Materials

The following supporting information can be downloaded at: https://www.mdpi.com/article/10.3390/plants11233315/s1, Table S1: Seed counts in each retrieval campaign; Table S2: Variation in number of seeds between t1 and t5 retrieval campaigns; Table S3: Analysis of variance on the number of seeds collected in the five retrieval campaigns (from t1 to t5); Table S4: Results of the GLM regression on seed counts; Table S5: Seed morphometric data; Table S6: Analysis of variance results; Table S7: Swelling seeds comparison with unmodified seeds; Table S8: Heavy metal concentration levels in the tailing dump; Table S9: Rainfall and soil moisture data measured in the study area; Figure S1: Seed count in each retrieval campaign in the different sowing treatments; Figure S2: Analysis of variance results on morphometric parameters measured in the different retrieval campaigns and in the different sowing treatments; Figure S3: Packet building; Figure S4: Developmental stages of *Epipactis helleborine* seedlings; Figure S5: Comparison between unmodified seed and developing seeds.
