# Supplementary material for: Seed Bank Conservation and Incipient Seed Development in Orchids Colonizing Mining Wastes: Results of a Field Pilot Experiment
Source: Plants (Basel). 2022 Dec 1;11(23):3315. doi: 10.3390/plants11233315 (PMC9740175; doi:10.3390/plants11233315)
Supplement: Supplementary file 1 [file plants-11-03315-s001.zip › plants-2065568-supplementary.pdf]

Table S1. Seed counts in each retrieval campaign**.**

The number of seed retrieved in each sowing treatment during the experiment (from t1 to t5) is reported as mean value (n=3) ± standard deviation (SD).

Retrieval campaign	Sowing treatment	Seed number (mean ± SD)
t1	*PP in PP*	283.33 ± 34.00
	PP in NP	269.67 ± 22.19
	NP in NP	255.00 ± 7.55
	NP in PP	276.00 ± 41.15
t2	*PP in PP*	277.33 ± 25.54
	PP in NP	255.00 ± 7.21
	NP in NP	322.00 ± 17.35
	NP in PP	275.00 ± 43.71
t3	*PP in PP*	295.00 ± 17.44
	PP in NP	278.33 ± 35.70
	NP in NP	318.00 ± 47.47
	NP in PP	307.00 ± 31.43
t4	*PP in PP*	271.33 ± 34.31
	PP in NP	233.00 ± 1.00
	NP in NP	251.00 ± 18.36
	NP in PP	277.33 ± 44.23
t5	*PP in PP*	282.33 ± 15.95
	PP in NP	236.67 ± 42.03
	NP in NP	267.67 ± 18.93
	NP in PP	279.00 ± 53.00

Table S2. Variation in number of seeds between t1 and t5 retrieval campaigns**.**

The number of seeds collected during the first and the last of the retrieval campaigns were tested to significantly differ between each other.

Sowing treatment	Test	Test statistics	Degree of freedom	p-value
PP in PP	t-test	0.046	4	0.965
PP in NP	t-test	1.203	4	0.295
NP in NP	t-test	-1.076	4	0.342
NP in PP	t-test	-0.077	4	0.942

Table S3. Analysis of variance on the number of seeds collected in the five retrieval campaigns (from t1 to t5).

Sowing treatment	Test	Test statistics	Degree of freedom	p-value	Effect size
PP in PP	ANOVA	0.324	4	0.856	0.36
PP in NP	ANOVA	1.651	4	0.237	0.81
NP in NP	ANOVA	5.431	4	0.014	1.47
NP in PP	ANOVA	0.295	4	0.874	0.34

Table S4. Results of the GLM regression on seed counts.

Summary of the GLM quasi-Poisson regression implemented on seed count in the sowing treatments (first column). Time was chosen as explanatory variable (second column) the estimated effect and the standard error (SE) are reported on the third and fourth columns respectively. The fifth column reports effects' *p*-values.

Sowing treatment	Retrieval campaign	Estimate	SE	*p*-value
PP in PP	Intercept	5.65	0.35	<0.001
	t2	-0.02	0.05	0.66
	t3	0.04	0.05	0.40
	t4	-0.04	0.05	0.38
	t5	-0.004	0.05	0.94
PP in NP	intercept	5.60	0.06	<0.001
	t2	-0.06	0.08	0.52
	t3	0.03	0.08	0.71
	t4	-0.15	0.09	0.12
	t5	-0.13	0.09	0.16
NP in NP	intercept	5.54	0.05	<0.001
	t2	0.23	0.07	0.009
	t3	0.22	0.07	0.01
	t4	-0.02	0.08	0.84
	t5	0.05	0.07	0.53
NP in PP	intercept	5.62	0.09	<0.001
	t2	-0.004	0.13	0.99
	t3	0.11	0.12	0.41
	t4	0.005	0.127	0.97
	t5	0.01	0.13	0.93

Table S5. Seed morphometric data**.**

Morphometric parameters (first and fifth columns) measured in each sowing treatment (second and sixth columns) in the different retrieval campaigns (third and seventh columns) are reported as mean value ± SE (fourth and eighth columns). *PP in PP* indicates metallicolous seeds sowed in the tailing dump; *PP in NP* indicates metallicolous seeds sowed in the control site; *NP in NP* indicates control seeds sowed in the control site; *NP in PP* indicates control seeds sowed in the tailing dump.

Parameter	***Sowing treatment***	***Retrieval campaign***	***Mean ± SE (mm/mm²)***	***Parameter***	***Sowing treatment***	***Retrieval campaign***	***Mean ± SE (mm/mm²)***
Coat width	*PP in PP*	t1	0.25 ± 0.011	Embryo width	*PP in PP*	t1	0.119 ± 0.004
		t2	0.29 ± 0.009			t2	0.127 ± 0.003
		t3	0.33 ± 0.016			t3	0.135 ± 0.003
		t4	0.26 ± 0.012			t4	0.130 ± 0.004
		t5	0.30 ± 0.018			t5	0.141 ± 0.004
	PP in NP	t1	0.28 ± 0.011		*PP in NP*	t1	0.123 ± 0.007
		t2	0.29 ± 0.011			t2	0.128 ± 0.005
		t3	0.28 ± 0.018			t3	0.134 ± 0.007
		t4	0.28 ± 0.015			t4	0.138 ± 0.005
		t5	0.27 ± 0.013			t5	0.133 ± 0.005
	NP in NP	t1	0.26 ± 0.012		*NP in NP*	t1	0.145 ± 0.009
		t2	0.26 ± 0.008			t2	0.135 ± 0.004
		t3	0.26 ± 0.009			t3	0.125 ± 0.004
		t4	0.28 ± 0.010			t4	0.132 ± 0.008
		t5	0.28 ± 0.014			t5	0.127 ± 0.006
	NP in PP	t1	0.23 ± 0.013		*NP in PP*	t1	0.136 ± 0.007
		t2	0.24 ± 0.009			t2	0.129 ± 0.006
		t3	0.25 ± 0.011			t3	0.123 ± 0.008
		t4	0.27 ± 0.008			t4	0.132 ± 0.005
		t5	0.27 ± 0.02			t5	0.135 ± 0.003
Coat length	*PP in PP*	t1	1.16 ± 0.039	Embryo length	*PP in PP*	t1	0.25 ± 0.008
		t2	1.11 ± 0.026			t2	0.24 ± 0.005
		t3	1.07 ± 0.031			t3	0.23 ± 0.007
		t4	1.00 ± 0.056			t4	0.22 ± 0.006
		t5	1.07 ± 0.06			t5	0.24 ± 0.005
	PP in NP	t1	1.01 ± 0.065		*PP in NP*	t1	0.24 ± 0.008
		t2	1.08 ± 0.039			t2	0.23 ± 0.007
		t3	1.05 ± 0.041			t3	0.23 ± 0.007
		t4	1.09 ± 0.066			t4	0.22 ± 0.004
		t5	1.03 ± 0.059			t5	0.23 ± 0.007
	NP in NP	t1	0.90 ± 0.048		*NP in NP*	t1	0.22 ± 0.01
		t2	0.88 ± 0.040			t2	0.21 ± 0.008
		t3	0.86 ± 0.062			t3	0.20 ± 0.012
		t4	0.94 ± 0.055			t4	0.23 ± 0.01
		t5	1.00 ± 0.045			t5	0.22 ± 0.008
	NP in PP	t1	0.88 ± 0.042		*NP in PP*	t1	0.23 ± 0.01
		t2	0.92 ± 0.031			t2	0.23 ± 0.005
		t3	0.96 ± 0.058			t3	0.23 ± 0.01
		t4	0.90 ± 0.053			t4	0.23 ± 0.008
		t5	0.81 ± 0.040			t5	0.24 ± 007
Coat area	*PP in PP*	t1	0.23 ± 0.013	Embryo area	*PP in PP*	t1	0.023 ± 0.001
		t2	0.24 ± 0.009			t2	0.024 ± 0.001
		t3	0.26 ± 0.013			t3	0.025 ± 0.001
		t4	0.23 ± 0.011			t4	0.023 ± 0.001
		t5	0.23 ± 0.021			t5	0.026 ± 0.001
	PP in NP	t1	0.23 ± 0.012		*PP in NP*	t1	0.023 ± 0.002
		t2	0.23 ± 0.013			t2	0.024 ± 0.001

		t3	0.23 ± 0.019			t3	0.024 ± 0.002
		t4	0.23 ± 0.019			t4	0.024 ± 0.001
		t5	0.21 ± 0.017			t5	0.024 ± 0.002
	NP in NP	t1	0.19 ± 0.018		*NP in NP*	t1	0.025 ± 0.002
		t2	0.18 ± 0.013			t2	0.023 ± 0.001
		t3	0.17 ± 0.013			t3	0.020 ± 0.001
		t4	0.21 ± 0.019			t4	0.024 ± 0.002
		t5	0.22 ± 0.018			t5	0.022 ± 0.001
	NP in PP	t1	0.16 ± 0.011		*NP in PP*	t1	0.024 ± 0.002
		t2	0.17 ± 0.007			t2	0.024 ± 0.002
		t3	0.18 ± 0.008			t3	0.023 ± 0.003
		t4	0.19 ± 0.016			t4	0.024 ± 0.001
		t5	0.19 ± 0.018			t5	0.025 ± 0.001

Table S6. Analysis of variance results.

Each morphometric parameter (first column) was tested in each sowing treatment (second column) to vary during the experiment. Analysis carried out, together with the *post-hoc* test (if implemented) are reported in the third column while fourth and fifth columns report test results. Sixth column reports the *post-hoc* results. The couples of retrieval campaigns where data were reported to significantly vary are reported in the sixth column linked by the ~ symbol and are accompanied by the p-values of the respective *post-hoc* test, in this column NA indicates the non-applicability of *post-hoc* test due non-significant analysis of variance. Seventh and eighth columns report degrees of freedom and effect size of the analysis respectively. *PP in PP* indicates metallicolous seeds sowed in the tailing dump; *PP in NP* indicates metallicolous seeds sowed in the control site; *NP in NP* indicates control seeds sowed in the control site; *NP in PP* indicates control seeds sowed in the tailing dump.

Morphometric parameter	***Sowing treatment***	***Variance test / post-hoc test***	***Variance test F value / chi-squared***	***p-values***	***Post-hoc test results***	***Degrees of freedom***	***Effect size***
Coat Width	*PP in PP*	ANOVA / Tukey's	5.37	0.001	t3~t1, p-value = 0.002<br>t4~t3, p-value = 0.005	4	0.66
	PP in NP	Kruskall	1.76	0.78	NA	4	-0.05
	NP in NP	ANOVA	0.99	0.42	NA	4	0.29
	NP in PP	ANOVA	2.13	0.09	NA	4	0.43
Coat length	*PP in PP*	ANOVA	1.81	0.14	NA	4	0.09
	PP in NP	ANOVA	0.26	0.90	NA	4	0.15
	NP in NP	Kruskall	4.9	0.3	NA	4	0.02
	NP in PP	ANOVA	1.46	0.23	NA	4	0.35
Coat area	*PP in PP*	ANOVA / Tukey's	4.51	0.003	t4~t3, p-value = 0.006<br>t5~t4, p-value = 0.007	4	0.61
	PP in NP	ANOVA	0.22	0.92	NA	4	0.14
	NP in NP	ANOVA	1.37	0.25	NA	4	0.34
	NP in PP	Kruskall	2.38	0.67	NA	4	-0.03
Embryo Width	*PP in PP*	Kruskall / Pairwise U test	15.01	0.005	t1~t3, p-value = 0.042<br>t1~t5, p-value = 0.042<br>t2~t5, p-value = 0.042	4	0.22
	PP in NP	ANOVA	0.96	0.44	NA	4	0.29
	NP in NP	Kruskall	5.047	0.904	NA	4	0.02
	NP in PP	ANOVA	0.67	0.61	NA	4	0.24
Embryo Length	*PP in PP*	ANOVA	2.13	0.091	NA	4	0.42
	PP in NP	ANOVA	0.56	0.69	NA	4	0.22
	NP in NP	ANOVA	0.86	0.5	NA	4	0.27
	NP in PP	ANOVA	0.19	0.94	NA	4	0.13
Embryo Area	*PP in PP*	Kruskall	9.41	0.051	NA	4	0.11
	PP in NP	ANOVA	0.12	0.97	NA	4	0.29
	NP in NP	ANOVA	1.39	0.25	NA	4	0.15
	NP in PP	ANOVA	0.27	0.89	NA	4	0.15

Table S7. Swelling seeds comparison with unmodified seeds.

Observed swelling seeds were tested to present significantly greater size than non-swelling seeds observed in the same sowing treatment and retrieval campaign (first and second column respectively). Morphometric parameters are reported in the fourth column of the table, while fifth and sixth columns report test results (p-values are corrected by Bonferroni correction). *PP in PP* indicates metallicolous seeds sowed in the tailing dump; *PP in NP* indicates metallicolous seeds sowed in the control site; *NP in NP* indicates control seeds sowed in the control site; *NP in PP* indicates control seeds sowed in the tailing dump.

Sowing treatment	***Retrieval campaign***	***Morphometric parameter***	***Test***	***Test statistics***	***Degree of freedom***	***p-value***	***Adjusted p-value***	***Effect size***
PP in PP	t3 (n = 1)	Coat width	*t*-test	t = -2.4038	10	0.02	1.112^{-1}	0.72
		Coat length	*t*-test	t = 2.883	10	0.992	1.000	0.87
		Coat area	*t*-test	t = -1.465	10	0.087	5.213^{-1}	0.44
		Embryo width	U test	W = NA	9	0.002	9.720^{-3}	0.90
		Embryo length	*t*-test	t = -1.991	10	0.037	2.236^{-1}	0.60
		Embryo area	*t*-test	t = -17.394	10	4.182^{-9}	2.509^{-8}	5.24
NP in NP	t3 (n = 4)	Coat width	*t*-test	t = -3.182	9	0.006	3.345^{-2}	1.01
		Coat length	*t*-test	t = 1.515	9	0.918	1.000	0.48
		Coat area	*t*-test	t = 1.882	9	0.954	1.000	0.59
		Embryo width	U test	W = NA	9	0.003	1.634^{-2}	0.89
		Embryo length	U test	W = NA	9	0.003	1.643^{-2}	0.89
		Embryo area	*t*-test	t = -32.573	9	5.958^{-11}	3.575^{-10}	10.30
NP in NP	T4 (n = 5)	Coat width	*t*-test	t = -2.775	10	0.010	5.888^{-2}	0.84
		Coat length	*t*-test	t = 2.1719	10	0.972	1.000	0.65
		Coat area	*t*-test	t = 1.5471	10	0.924	1.000	0.47
		Embryo width	*t*-test	t = -14.252	10	2.855^{-8}	1.713^{-7}	4.29
		Embryo length	*t*-test	t = -5.567	10	1.192^{-4}	7.152^{-4}	1.68
		Embryo area	*t*-test	t = -16.911	10	5.493^{-9}	3.296^{-8}	5.10
NP in PP	t4 (n = 1)	Coat width	*t*-test	t = -15.103	11	5.304^{-9}	3.182^{-8}	4.36
		Coat length	*t*-test	t = 1.8914	11	0.957	1.000	0.54
		Coat area	*t*-test	t = -3.8097	10	0.002	1.030^{-2}	1.15
		Embryo width	*t*-test	t = -9.868	11	4.22^{-7}	2.535^{-6}	2.849
		Embryo length	*t*-test	t = 6.5998	11	1.000	1.000	1.905
		Embryo area	*t*-test	t = -3.7491	11	0.002	9.642^{-3}	1.082

Table S8. Heavy metal concentration levels in the tailing dump. Heavy metal concentration levels in the tailing dump that hosts the contaminated population (*PP* in the text). Data are reported as mean values (mg g^{-1}) ± standard deviation. Data originally reported (in [14] in the main text).

Element	Concentration
**Fe**	55.98 ± 7.44
**Zn**	13.10 ± 2.71
**Pb**	5.21 ± 0.69
**Mn**	1.24 ± 0.06
**Cu**	0.79 ± 0.08

Table S9. Rainfall and soil moisture data measured in the study area.

Range of potential evapotranspiration data (third column) and range of precipitation (fourth column) measured during the retrieval campaigns reference months (second column). Data were obtained from climatic monitoring authority of Sardinia and were measured by the weather stations placed in the vicinities of the study area where the field phase of the experiment took place.

References:

Agenzia Regionale per la Protezione dell'Ambiente della Sardegna (Arpas), Dipartimento Specialistico Regionale MeteoClimatico 2021a. Riepilogo mensile meteorologico e agrometeorologico - Marzo 2021.

Agenzia Regionale per la Protezione dell'Ambiente della Sardegna (Arpas), Dipartimento Specialistico Regionale MeteoClimatico 2021b. Riepilogo mensile meteorologico e agrometeorologico - Maggio 2021.

Agenzia Regionale per la Protezione dell'Ambiente della Sardegna (Arpas), Dipartimento Specialistico Regionale MeteoClimatico 2020a. Riepilogo mensile meteorologico e agrometeorologico - Novembre 2020.

Agenzia Regionale per la Protezione dell'Ambiente della Sardegna (Arpas), Dipartimento Specialistico Regionale MeteoClimatico 2020b. Riepilogo mensile meteorologico e agrometeorologico - Dicembre 2020.

Agenzia Regionale per la Protezione dell'Ambiente della Sardegna (Arpas), Dipartimento Specialistico Regionale MeteoClimatico 2020c. Riepilogo mensile meteorologico e agrometeorologico - Gennaio 2021.

Retrieval campaign	***Reference month***	***Potential evapotranspiration (mm)***	***Rainfall (mm)***
t1	November (2020)	31 – 35	21 – 40
t2	December (2020)	23 – 24	31 – 40
t3	January (2021)	21 – 25	41 – 50
t4	March (2021)	61 – 65	0 – 5
t5	May (2021)	111 – 120	21 – 30

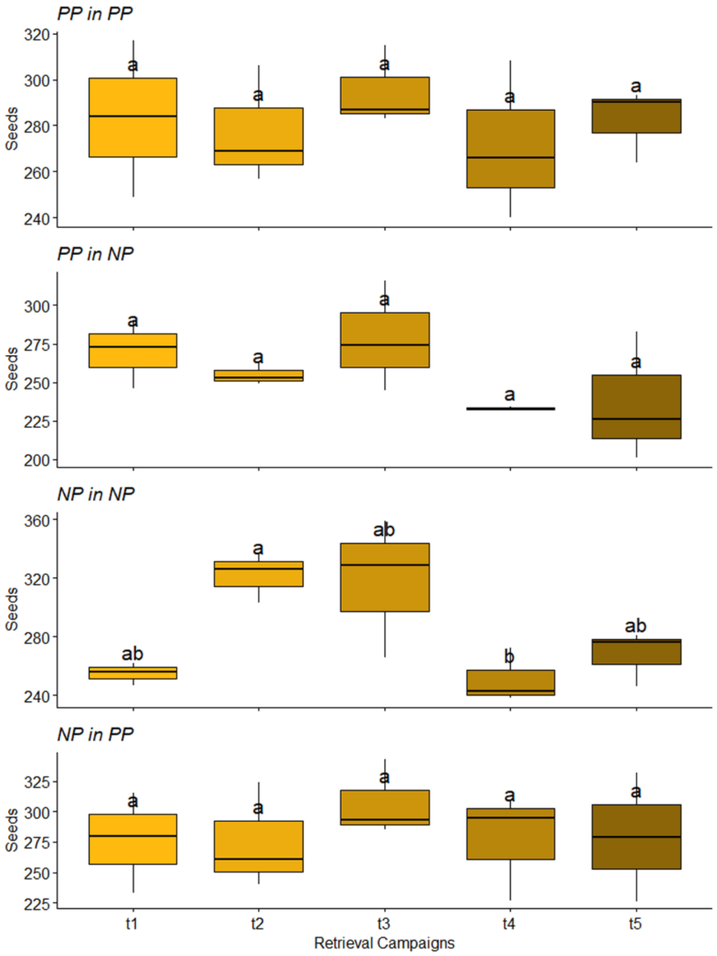

Figure S1. Seed count in each retrieval campaign in the different sowing treatments.

Each panel of the figure reports data from the different sowing treatments (PP in PP indicates metallicolous seeds sowed in the tailing dump; PP in NP indicates metallicolous seeds sowed in the control site; NP in NP indicates control seeds sowed in the control site; NP in PP indicates control seeds sowed in the tailing dump). Number of seeds is reported on the y axis, retrieval campaigns are reported on the x axis. The different retrieval campaigns are indicated by different colours. ANOVA results are reported above each series of boxplots as compact letter display. Level alfa at 0.05. Each boxplot reports 50% of the measured values (inside the box), comprised between the first quartile value (lower side of the box) and the third quartile value (upper side of the box), the median is indicated by the black line inside the box, while whiskers join the first and third quartiles with lower and higher measured value respectively (if present, outliers are reported as black dots).

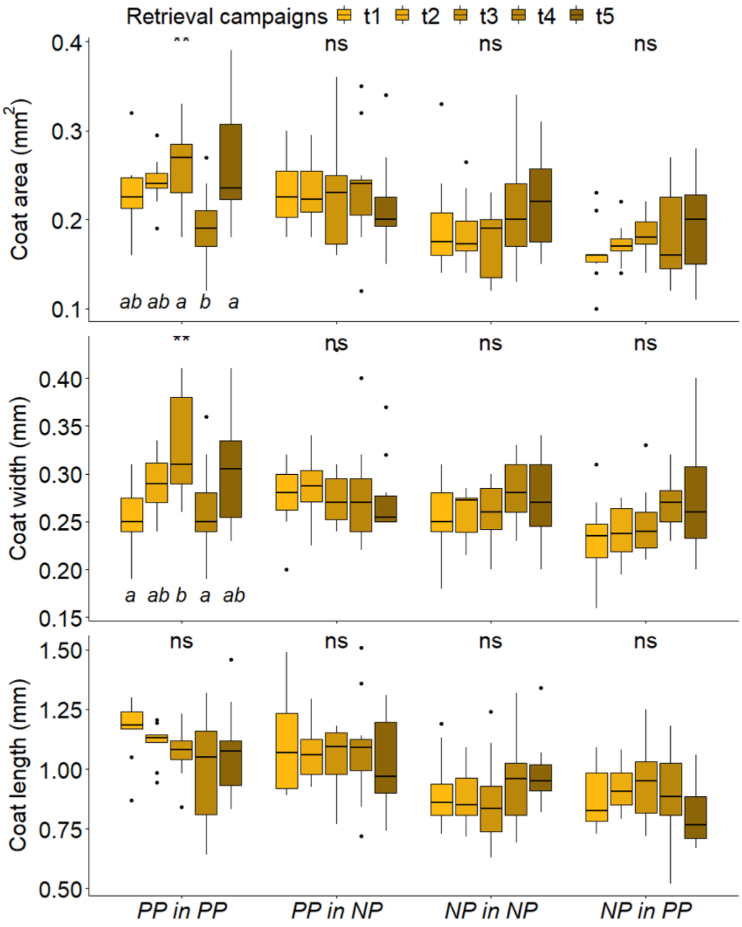

Figure S2. Analysis of variance results on morphometric parameters measured in the different retrieval campaigns and in the different sowing treatments.

Morphometric parameters are reported on the y axis, sowing treatments are reported on the x axis (PP in PP indicates metallicolous seeds sowed in the tailing dump; PP in NP indicates metallicolous seeds sowed in the control site; NP in NP indicates control seeds sowed in the control site; NP in PP indicates control seeds sowed in the tailing dump). Retrieval campaigns from t1 to t5 are indicated by different colours. ANOVA or Kruskall-Wallis test results are reported above each series of boxplots as follow: ns=non-significant; * $p<0.05$; ** $p<0.01$; *** $p<0.001$. Post-hoc results are reported by compact letter display under each boxplot. Level alfa at 0.05. Each boxplot reports 50% of the measured values (inside the box), comprised between the first quartile value (lower side of the box) and the third quartile value (upper side of the box), the median is indicated by the black line inside the box, while whiskers join the first and third quartiles with lower and higher measured value respectively (if present, outliers are reported as black dots).

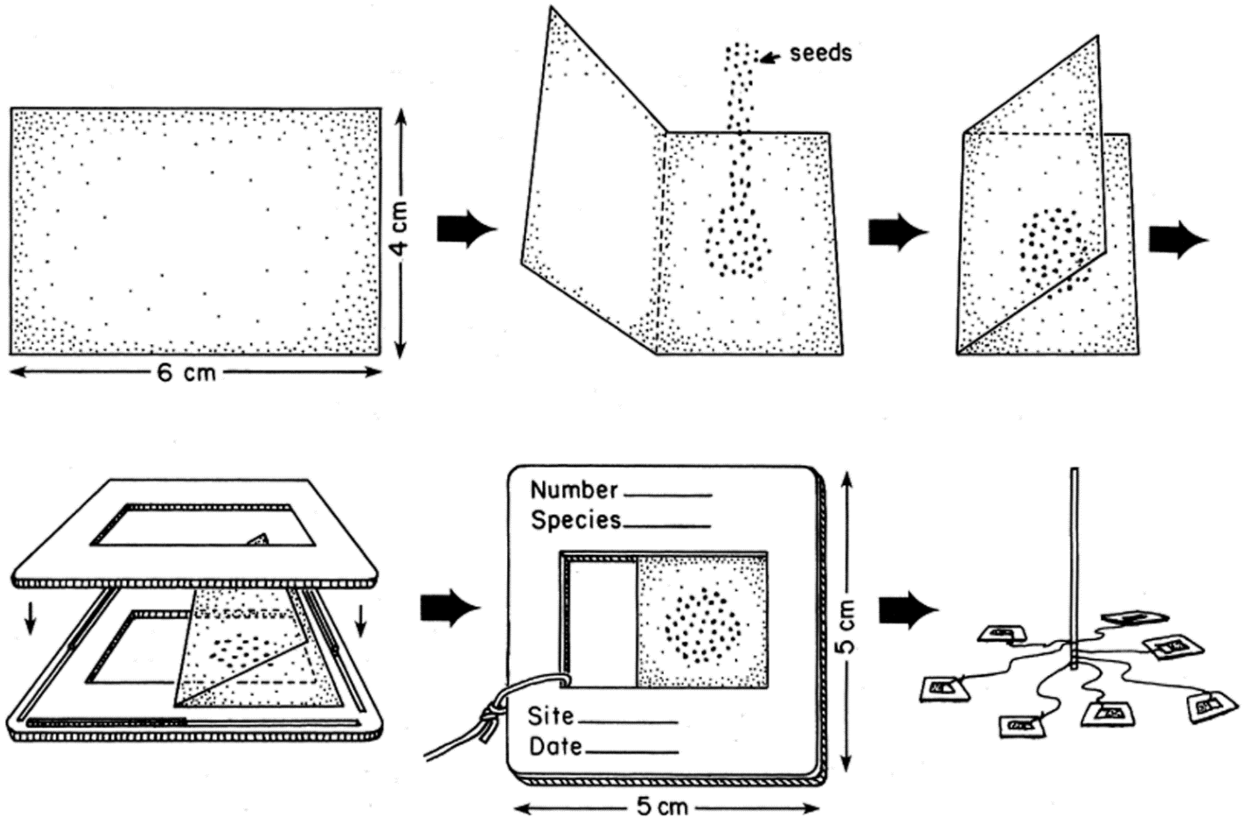

Figure S3. Packet building.
Packets used in the present study were built as reported in Rasmussen and Whigham (1993) (reported in the manuscript as [16]) with minor modifications.

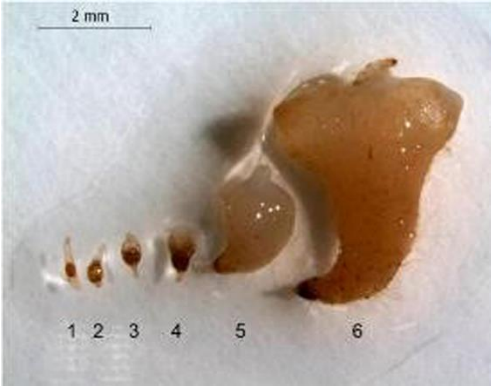

Figure S4. Developmental stages of *Epipactis helleborine* seedlings.

Epipactis helleborine is a closely related species of *Epipactis tremolsii*. In Těšitelová et al. (2012) (reported in the manuscript as [32]) are reported the initial developmental stages of the species from unmodified seed to branched seedling. "Stage 1: ungerminated seed; stage 2: swollen, non-mycorrhizal seedling; stage 3: small, oval-shaped mycorrhizal seedling; stage 4: pear-shaped seedling longer than 0.5 mm, a protocorm; stage 5: seedling with leaf primordium longer than 1 mm; and stage 6: branched seedling".

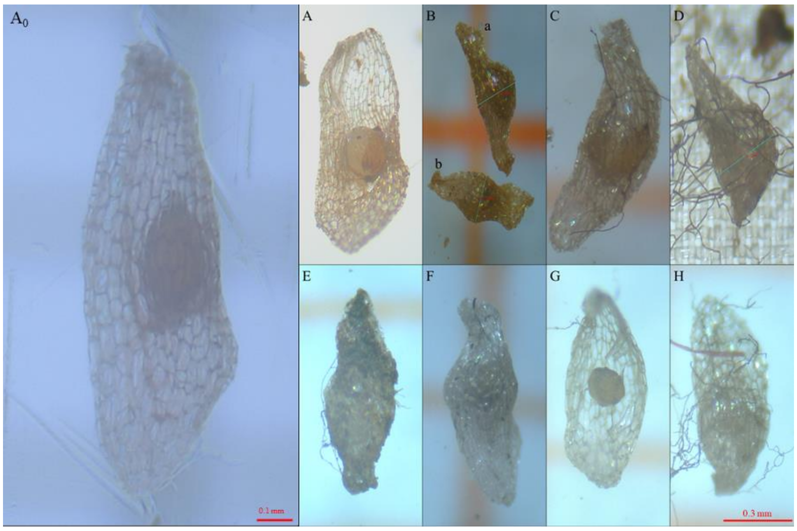

Figure S5. Comparison between unmodified seed and developing seeds.

In panel A0 an unmodified seed to be compared with some of the developing seeds observed during the experiment. A, B (a, b), C and D were collected on the third retrieval campaign while E, F, G and H were collected on the fourth. To notice the sensible increase in the embryo width in the passage from S1 to S2 developmental stage.
